# Elucidating the Role of Sirtuin 3 in Mammalian Oocyte Aging

**DOI:** 10.3390/cells13181592

**Published:** 2024-09-22

**Authors:** Pawel Kordowitzki

**Affiliations:** 1 Department of Preclinical and Basic Sciences, Faculty of Biological and Veterinary Sciences, Nicolaus Copernicus University, 87-100 Torun, Poland; p.kordowitzki@umk.pl; 2 Department of Gynecology Including Center of Oncological Surgery (CVK) and Department of Gynaecology (CBF), Charite, 13353 Berlin, Germany; 3 Department of Cell Biology, Harvard Medical School, Boston, MA 02115, USA

**Keywords:** SIRT3, sirtuin 3, oocyte, egg, embryo, mitochondria, aging, reproductive aging

## Abstract

The field of reproductive biology has made significant progress in recent years, identifying specific molecular players that influence oocyte development and function. Among them, sirtuin 3 (SIRT3) has attracted particular attention for its central role in mediating mitochondrial function and cellular stress responses in oocytes. So far, studies have demonstrated that the knockdown of SIRT3 leads to a decrease in blastocyst formation and an increase in oxidative stress within an embryo, underscoring the importance of SIRT3 in maintaining the cellular redox balance critical for embryonic survival and growth. Furthermore, the literature reveals specific signaling pathways, such as the SIRT3- Glycogen synthase kinase-3 beta (GSK3β) deacetylation pathway, crucial for mitigating oxidative stress-related anomalies in oocyte meiosis, particularly under conditions like maternal diabetes. Overall, the emerging role of SIRT3 in regulating oocyte mitochondrial function and development highlights the critical importance of understanding the intricate connections between cellular metabolism, stress response pathways, and overall reproductive health and function. This knowledge could lead to the development of novel strategies to support oocyte quality and fertility, with far-reaching implications for assisted reproductive technologies and women’s healthcare. This commentary aims to provide an overview of the importance of SIRT3 in oocytes by synthesizing results from a multitude of studies. The aim is to elucidate the role of SIRT3 in oocyte development, maturation, and aging and to identify areas where further research is needed.

## 1. Introduction

**Figure 1:**
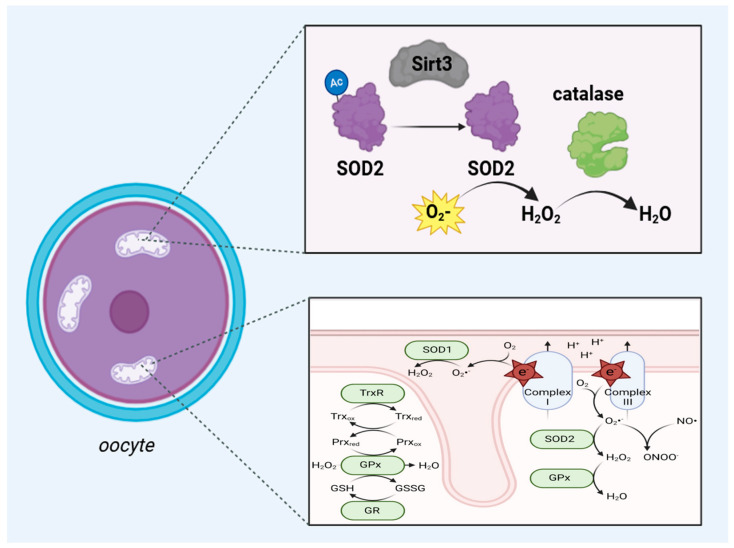
The upper part of this scheme shows the antioxidant activity of the SIRT3/SOD2/catalase axis in the mitochondria of oocytes. In the matrix of the mitochondria, upon the activity of SIRT3, the deacylation of SOD2 occurs, which is responsible for converting superoxide (O_2_) into hydrogen peroxide (H_2_O_2_), and catalase converts H_2_O_2_ into water. The lower panel shows a schematic drawing of ROS generation and inactivation in mitochondria. Most ROS originate from complexes I and III of the electron transport chain when electrons react with electrophilic oxygen molecules to produce superoxide (free radical O_2_). Superoxide is split into hydrogen peroxide (H_2_O_2_) by SOD1 in the mitochondrial intermembrane space and SOD2 in the mitochondrial matrix. Hydrogen peroxide is then further inactivated by the cooperation of glutathione peroxidase (GPx), peroxiredoxin (Prx), and thioredoxin (Trx) family members (TrxR: thioredoxin reductase 2; GR: glutathione reductase) using nicotinamide adenine dinucleotide phosphate hydrogen (NADPH) as the reducing equivalent Abbreviations: NO = nitric oxide; ONOO^−^ = peroxynitrite; SOD1 = superoxide dismutase type 1; SOD2 = superoxide dismutase type 2; GSH = glutathione, GSSG = Glutathione disulfide; Trxox = thioredoxin oxidation, Trxred = thioredoxin reduction; Prxox = peroxiredoxin oxidation; Prxred = peroxiredoxin reduction. This figure was created based on the tools provided by Biorender.com (https://biorender.com/; accessed on 24 August 2024) and adapted from [14].

The field of reproductive biology has made substantial strides in recent years, pinpointing specific molecular players that influence oocyte development and function. Among these, sirtuin 3 (SIRT3) has garnered particular attention for its pivotal role in mediating mitochondrial function and cellular stress responses within oocytes [1,2]. Sirtuin 3 is a member of the sirtuin family of enzymes, which promote a wide range of cellular processes, including metabolism, apoptosis, and aging [3,4]. As a nicotinamide adenine dinucleotide (NAD^+^)-dependent deacetylase, SIRT3 primarily targets mitochondrial proteins, modifying their activity to optimize energy production and utilization. One of the key functions of SIRT3 is its ability to enhance fatty acid oxidation, the tricarboxylic acid (TCA)cycle, and the urea cycle, thereby promoting more efficient energy metabolism [5]. This is achieved through the deacetylation and activation of numerous mitochondrial metabolic enzymes, such as those involved in the TCA cycle (Figure 1) [6,7]. SIRT3 also regulates the activity of enzymes involved in glutamine metabolism, amino acid catabolism, and antioxidant defense, further demonstrating its wide-ranging impact on mitochondrial function [8]. The activity of SIRT3 is closely linked to the levels of NAD^+^ within the mitochondria [9]. Recent studies have shown that increasing NAD^+^ levels, either through dietary interventions or pharmacological approaches, can enhance SIRT3 activity and exert beneficial effects on cardiac and renal health [10]. For example, the administration of NAD^+^ precursors has been observed to improve cardiac function and reduce the risk of age-related diseases, potentially mediated by the activation of SIRT3 and other sirtuins [11,12]. Conversely, downregulation or impairment of SIRT3 has been associated with various pathological conditions, including metabolic disorders, neurodegenerative diseases, and cancer [3]. Previous research has provided strong evidence that the knockdown of SIRT3 leads to a decrease in blastocyst formation and an increase in oxidative stress within the embryo, underscoring the importance of SIRT3 in maintaining the cellular redox balance critical for embryonic survival and growth [13,14].

Furthermore, specific signaling pathways, such as the SIRT3-GSK3β deacetylation pathway, have been described as being crucial for mitigating oxidative stress-related anomalies during oocyte meiosis, particularly under conditions like maternal diabetes [15]. Studies indicate that SIRT3’s modulation by compounds such as dihydromyricetin and curcumin through the activation of the peroxisome proliferator-activated receptor co-activator-1α (PGC)-1α/SIRT3 signaling pathway holds potential therapeutic value for enhancing oocyte quality and fertility [16]. Additionally, research has highlighted the compensatory upregulation of SIRT1 in Sirt3-null mice, suggesting a sophisticated interplay among sirtuin family members that aids in maintaining mitochondrial function under duress [17]. Other research has described the expression of SIRT3 in oocytes under different physiological and pathological conditions, touching upon reproductive aging, obesity, and polycystic ovary syndrome (PCOS) [18,19,20,21]. Evidence from the literature suggests that SIRT3 levels are variable and respond adaptively to different stress conditions, making it a promising target for therapeutic interventions aimed at improving reproductive outcomes in women facing infertility challenges. For example, SIRT3 expression was found to be upregulated in response to oxidative stress, underscoring its role in supporting ovarian physiology and development [22,23,24]. Despite the progress in understanding SIRT3’s role, there remain substantial gaps in the knowledge regarding its specific mechanisms of action and interactions with other sirtuins. There is an ultimate need for further exploration into how SIRT3’s regulatory functions can be harnessed to mitigate conditions like reproductive aging and metabolic disorders that impair oocyte quality [22]. Notably, studies on the impact of SIRT3 on mitochondrial function in oocytes, including its effects on spindle assembly and chromosomal stability, may offer insights into novel therapeutic avenues. By addressing the questions above, this commentary aims to synthesize the current understanding of SIRT3’s multifaceted role in oocyte development, maturation, and aging. It seeks to provide a cohesive overview that highlights existing findings and identifies prospective research directions crucial for advancing reproductive biology and aging to improve fertility outcomes, ultimately contributing to a broader understanding of female reproductive health in mammalian species.

## 2. Sirtuin 3 and Aging

**Figure 2:**
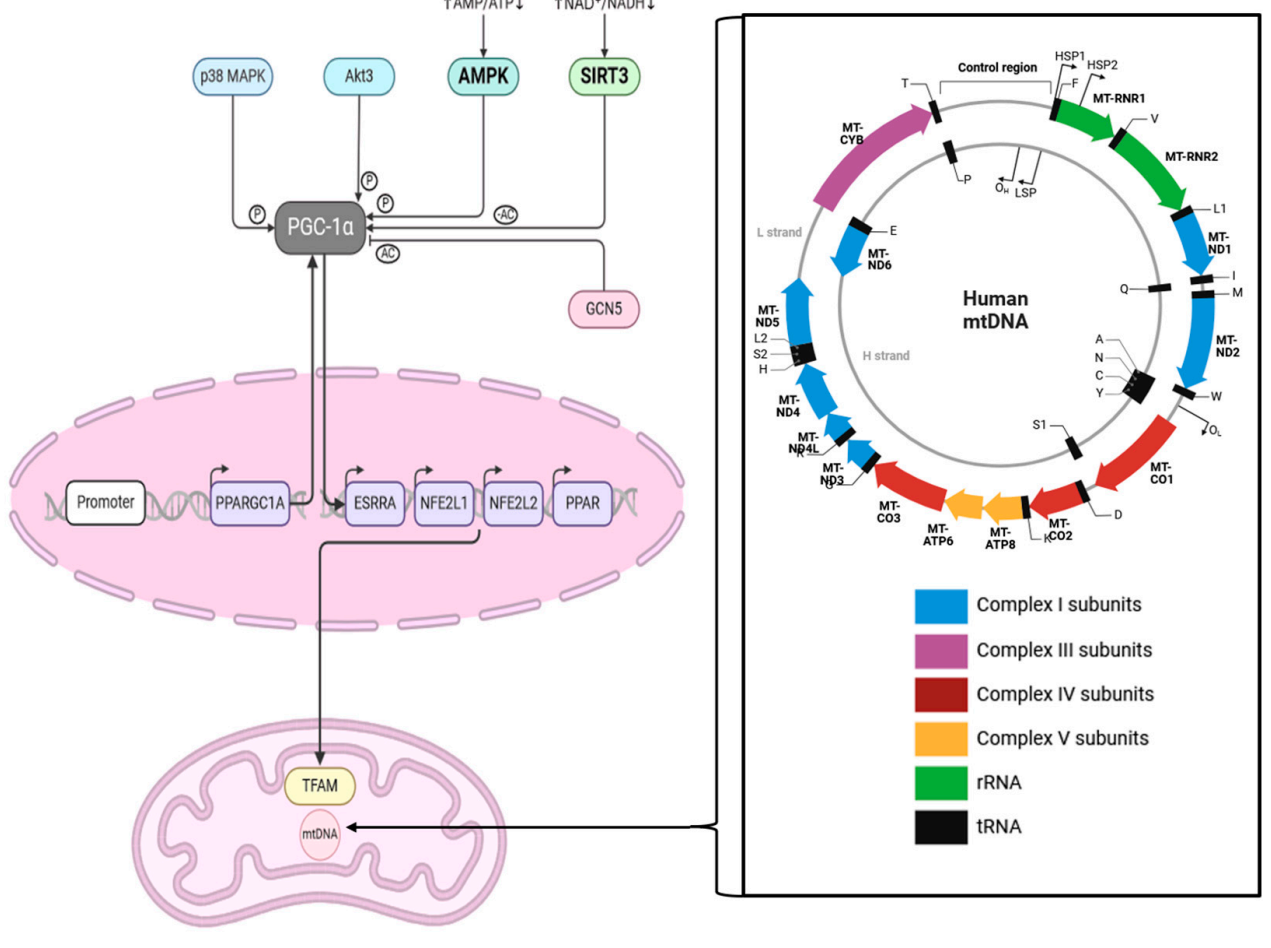
This scheme shows the interaction between SIRT3 and PGC1α and the cascade influencing TFAM in mitochondria. The zoomed-in box on the right-hand side shows the human mitochondrial genome, which is 16,569 bp long. Abbreviations: MAPK = mitogen-activated protein kinase, Akt3 = RAC-gamma serine/threonine-protein kinase, AMPK = AMP-activated protein kinase, AMP = Adenosine monophosphate, NAD = Nicotinamide adenine dinucleotide, NADH = nicotinamide adenine dinucleotide+ hydrogen, PPARGC1A = Peroxisome proliferator-activated receptor gamma coactivator 1-alpha, ESRRA = Estrogen receptor-related receptor, NFE2L1 = nuclear factor erythroid 2 (NF-E2)-related factor 1, NFE2L2 = nuclear factor erythroid 2 (NF-E2)-related factor 2, PPAR = Peroxisome proliferator-activated receptor, TFAM = mitochondrial transcription factor A, Ac = acetylation; P = phosphorylation. This figure was created based on the tools provided by Biorender.com (https://biorender.com/; accessed 24 August 2024) and adapted from [36,37].

Aging is a complex and multifaceted process that involves the gradual deterioration of various physiological functions, leading to an increased susceptibility to various age-related diseases and, ultimately, mortality. Regulating aging and extending lifespan has been a subject of intense research. Among the seven mammalian sirtuins (SIRT1–SIRT7), SIRT3 has emerged as a crucial player in the regulation of metabolism and the aging process [25,26]. SIRT3 is primarily localized in the mitochondria and has been shown to modulate the activity of numerous metabolic enzymes, thereby enhancing mitochondrial function and energy production, as shown, among others, for mitochondrial functional parameters such as basal respiration and ATP production [3,27]. Overexpression of SIRT3 increases SOD2 activity, increases the expression of mitochondrial DNA-encoded genes, and alters mitochondrial dynamics [27]. Furthermore, SIRT3 overexpression also causes increased expression of mitochondrial biogenesis genes and proteins such as PGC-1α and mitochondrial transcription factor A (TFAM) (Figure 2). Recent research data generated from cardiomyoblasts confirm the alterations mentioned before [27]. Importantly, SIRT3 expression and activity have been observed to decline with age, suggesting a potential link between SIRT3 and the aging phenotype [28]. The role of SIRT3 in regulating metabolism and mitochondrial function has led to the hypothesis that it may also cause a modulation of lifespan and healthspan. Indeed, several studies have reported that increased SIRT3 expression or activity is associated with an extended lifespan and the delayed onset of age-related diseases in various model organisms [29,30]. For instance, calorie restriction, a well-known intervention that can extend lifespan, has been shown to upregulate SIRT3 expression and activity [31]. Moreover, pharmacological activation of SIRT3 has been demonstrated to mimic the beneficial effects of calorie restriction, suggesting that SIRT3 may be a key mediator of the lifespan-extending effects of this dietary intervention [32]. In addition to its role in metabolism and mitochondrial function, SIRT3 has also been linked to the regulation of other cellular processes that are relevant to aging, such as the cellular stress response, genomic stability, and stem cell function [33,34,35]. In conclusion, sirtuin 3 has emerged as a crucial player in regulating metabolism, mitochondrial function, and the aging process. SIRT3’s ability to deacetylate and activate a wide range of metabolic enzymes, as well as its role in maintaining mitochondrial integrity and function, make it a promising target for developing interventions to promote healthy aging and longevity.

## 3. The Role of Sirtuin 3 in Oocyte Development

In the context of oocyte development, SIRT3 has been found to impact mitochondrial function and oocyte quality. Oocytes have a high metabolic demand due to the need to support early embryonic development, and SIRT3 helps to ensure that the oocyte’s mitochondria are functioning optimally, meaning providing proper energy, especially during oocyte maturation and fertilization. In consequence, the quality of oocytes’ mitochondria impacts the quality of the oocytes [38,39]. Aging-related mitochondrial dysfunction reflects suboptimal metabolic, redox, and calcium signaling processes in oocytes. Disruption of SIRT3 has been linked to impaired oocyte quality, reduced blastocyst development, and an increased incidence of aneuploidy [23,40]. Furthermore, the activity of SIRT3 is regulated by various mechanisms, including transcriptional control, post-translational modifications, and interactions with other proteins [41]. Upregulation of NAD^+^ biosynthesis, which is a key cofactor for SIRT3 activity, has been shown to dramatically extend lifespan in yeast, suggesting that modulating SIRT3 activity could be a promising approach for improving oocyte quality and fertility [42]. Overall, the emerging role of SIRT3 in regulating oocyte mitochondrial function and development highlights the importance of understanding the intricate connections between metabolism, stress response, and reproductive health [43].

The development of oocytes is a complex process characterized by intricate cellular and molecular changes that ensure the proper formation and maturation of these gametes. Central to this process is the mitochondrial sirtuin SIRT3, which has been recognized for its critical functions in regulating mitochondrial activity, metabolism, and stress responses within oocytes. Oocyte development, also known as oogenesis, encompasses the maturation of primordial germ cells into fully developed oocytes capable of undergoing fertilization [44,45]. This developmental trajectory is orchestrated by a series of tightly regulated stages, including the formation of primary oocytes, growth phases, and the maturation process that culminates in the meiotic division necessary for fertilization [45]. Throughout these stages, mitochondrial function is important, providing proper energy, such as ATP, and metabolic support required for oocyte growth and maturation. It is within this mitochondrial landscape that SIRT3 emerges as a key regulator [46]. SIRT3 functions predominantly through its deacetylase activity on various mitochondrial proteins, thereby modulating their function and stability. One of the most well-documented targets of SIRT3 is superoxide dismutase 2 (SOD2), an enzyme critical for detoxifying reactive oxygen species (ROS) [47,48,49]. By deacetylating and activating SOD2, SIRT3 enhances the antioxidant defense mechanism within oocytes, mitigating oxidative stress and ensuring cellular homeostasis (Figure 1) [50]. This function is especially crucial given that oxidative stress is a significant contributor to oocyte aging and quality degradation [22,46]. As previously briefly mentioned, studies have highlighted the upregulation of SIRT3 in oocytes exposed to oxidative stress [24]. The impact of SIRT3 on mitochondrial function in oocytes extends beyond its antioxidant activity. SIRT3 also influences mitochondrial dynamics, including processes such as mitochondrial biogenesis, fusion, and fission. These processes are essential for the maintenance of mitochondrial integrity and function, particularly during the energy-intensive phases of oocyte development. By modulating the acetylation status of key enzymes involved in the TCA cycle, SIRT3 promotes efficient ATP production, ensuring that oocytes have adequate energy reserves for their developmental and maturation processes [51]. The positive regulation of genes such as Gdf9 and Bmp15 by SIRT3 underscores its involvement in follicle development and the hormonal environment necessary for successful oocyte maturation [52]. These findings suggest that SIRT3 acts at the cellular level to maintain mitochondrial function and supports overall ovarian health and reproductive potential. In conclusion, the insights garnered from recent studies pave the way for potential therapeutic applications with regard to female reproductive aging.

## 4. Influence of SIRT3 on Oocyte Maturation

Oocyte maturation is a multi-stage process encompassing a transition from the germinal vesicle stage through meiotic progression, concluding with the formation of a mature oocyte ready for fertilization. Within this intricate biological process, SIRT3 emerges as a fundamental orchestrator, influencing various stages of oocyte maturation through its regulatory roles in mitochondrial function and cellular metabolism [53]. The stages of oocyte maturation are characterized by a series of tightly regulated cellular events. Initially, oocytes are arrested at the germinal vesicle stage within the ovarian follicles. Upon receiving appropriate hormonal signals, such as luteinizing hormone (LH) surge, the oocytes resume meiosis, progressing through germinal vesicle breakdown (GVBD), metaphase I, anaphase I, and ultimately arresting at metaphase II until fertilization [54,55,56]. During these stages, the role of mitochondria becomes increasingly critical as they provide the energy necessary for successful meiotic progression and cytoplasmic maturation [15,46]. SIRT3 exerts its influence on oocyte maturation primarily through the modulation of mitochondrial activity and the cellular redox state. Key signaling pathways regulated by SIRT3 include the SIRT3-GSK3β deacetylation pathway and the PGC-1α/SIRT3 axis, which are integral in maintaining mitochondrial function and oxidative stress response [57]. The deacetylation of GSK3β by SIRT3 contributes to the metabolic regulation and energy homeostasis within oocytes [15,58,59]. Moreover, the activation of the PGC-1α/SIRT3 pathway promotes mitochondrial biogenesis and enhances the antioxidative defense, thus ensuring the fidelity of oocyte maturation under varying physiological conditions [59]. Comparative studies on various SIRT isoforms highlight the unique and indispensable role of SIRT3 in the oocyte maturation process. While SIRT1 and SIRT5 also contribute to cellular homeostasis and redox regulation, the specific deacetylation targets and mitochondrial localization of SIRT3 position it as a central regulator in this context. For example, SIRT1 is more broadly involved in nuclear transcriptional regulation, whereas SIRT5’s function overlaps with SIRT3 but is less specialized in the context of mitochondria. These distinctions underscore why SIRT3 is particularly suited to address the energy demands and oxidative challenges of maturing oocytes [60,61,62]. Additionally, comparative insights reveal that SIRT3’s role extends beyond simple metabolic regulation. In conclusion, SIRT3’s influence on oocyte maturation is multifaceted, spanning metabolic regulation, mitochondrial function, and gene expression modulation. Its critical role is evident across various stages of maturation, from germinal vesicle breakdown to metaphase II arrest. The experimental and comparative studies reviewed in this chapter elucidate the essential functions of SIRT3 and corroborate its position as a central figure in the complex oocyte maturation process. Consolidating these insights, it becomes apparent that SIRT3 supports the intrinsic energy requirements and antioxidative defenses of oocytes and contributes to a broader regulatory network that ensures successful maturation.

## 5. The Relevance of SIRT3 for Mitochondria

Mitochondria, often cited as the powerhouses of the cell, are indispensable to a multitude of cellular processes, including energy production, the regulation of metabolic pathways, and the maintenance of cellular health [63]. Metabolism is a cornerstone of cellular function, providing the necessary energy for numerous biological processes. Noteworthily, only maternal mitochondria are transferred to the offspring; in other words, a newly formed embryo receives the mitochondria solely from the oocyte [46,64]. Sirtuin 3 advances several aspects of mitochondrial metabolism, ensuring efficient energy production and maintaining metabolic homeostasis [8,65,66]. Sirt3 is intricately involved in oxidative phosphorylation, a primary mechanism for ATP production within the mitochondria. Sirt3 exerts its influence by deacetylating and activating critical enzymes within the TCA cycle and the electron transport chain (ETC) [67,68]. These enzymes include acetyl-CoA synthetase 2 and glutamate dehydrogenase, whose activities are essential for the efficient functioning of the TCA cycle and subsequent ATP generation (Figure 1) [69]. By enhancing the efficiency of these pathways, Sirt3 ensures that the mitochondria can produce ATP at a rate that meets cellular energy demands. Beyond its role in oxidative phosphorylation, Sirt3 significantly contributes to fatty acid oxidation, another vital source of energy, especially during periods of fasting or caloric restriction [70,71]. In muscle cells and hepatocytes, Sirt3 interacts with peroxisome proliferator-activated receptor co-activator-1α (PGC-1α), which regulates adaptive thermogenesis, gluconeogenesis, and mitochondrial biogenesis. This interaction signifies Sirt3’s critical role in modulating energy production through fatty acid oxidation [72]. The deacetylation of enzymes involved in this metabolic pathway by Sirt3 highlights its adaptability in response to varying cellular energy needs. Sirt3’s impact on the electron transport chain is profound. The ETC is responsible for creating a proton gradient across the mitochondrial membrane, which drives ATP synthesis through oxidative phosphorylation [46]. Sirt3 activates several key enzymes within the respiratory chain, such as the succinate dehydrogenase complex, ensuring proper electron flow and maintaining the efficiency of oxidative phosphorylation [73]. This regulation is vital for cellular energy homeostasis, as any disruption in the ETC can lead to decreased ATP production and increased oxidative stress [46]. In addition to modulating energy production pathways, Sirt3 participates in regulating reactive oxygen species within the mitochondria [74]. ROS are by-products of metabolic processes and can cause significant cellular damage if not adequately controlled [46]. Sirt3 mitigates oxidative stress by activating antioxidant enzymes such as manganese superoxide dismutase (MnSOD) and catalase [75]. These enzymes neutralize ROS, thereby protecting the cell from oxidative damage and related diseases [76]. Sirt3’s ability to maintain ROS levels within a balanced range underscores its importance in preserving mitochondrial and cellular health. In conclusion, Sirt3’s role in mitochondrial metabolism is complex, encompassing the regulation of fatty acid oxidation and the electron transport chain. Its actions ensure efficient energy production and management of reactive oxygen species, which are crucial for cellular health, aging, and longevity. By modulating these metabolic pathways, Sirt3 maintains mitochondrial integrity and function, supporting overall cellular homeostasis and resilience against metabolic stress and diseases.

## 6. Function of SIRT3 during Mitochondrial Biogenesis

Mitochondrial biogenesis is the process by which cells increase their mitochondrial mass and copy numbers to meet heightened energy demands and maintain metabolic homeostasis [36]. Sirtuin 3 reflects an instrumental role in this process through its influence over a range of signaling pathways and regulatory factors, as well as in oocytes [77]. This chapter explores the mechanisms underlying mitochondrial biogenesis, the interaction between Sirt3 and key pathways such as the peroxisome proliferator-activated receptor γ coactivator 1α (PGC-1α) and 5’AMP-activated protein kinase (AMPK), Sirt3’s role in stress responses, and its impact on mitochondrial DNA (Figure 2) maintenance. The mechanisms of mitochondrial biogenesis are grounded in the activation of several transcription factors and co-activators, foremost among them being peroxisome proliferator-activated receptor gamma coactivator 1-alpha (PGC-1α) [36]. PGC-1α serves as a central transcriptional co-activator that stimulates the expression of genes involved in mitochondrial DNA replication, transcription, and protein synthesis. Sirt3 exerts its effect by deacetylating and activating PGC-1α, thereby enabling it to enhance the transcription of nuclear respiratory factors (NRF1 and NRF2) [78] and mitochondrial transcription factor A (TFAM), which orchestrate the synthesis of new mitochondria (Figure 2) [37,79]. This interaction is vital as it lays the foundational steps for mitochondrial biogenesis. Sirt3’s regulation of PGC-1α extends further into controlling oxidative stress. As previously mentioned, by deacetylating PGC-1α, Sirt3 increases the activity of antioxidant enzymes. This conjugate regulation of oxidative stress and mitochondrial biogenesis highlights Sirt3’s centrality in maintaining cellular health. AMP-activated protein kinase (AMPK) also contributes significantly to mitochondrial biogenesis, acting as an energy sensor that activates in response to low cellular energy levels (Figure 2) [80]. Upon activation, AMPK phosphorylates and activates PGC-1α, thereby promoting mitochondrial biogenesis. Sirt3 influences this pathway by enhancing AMPK activity, which further potentiates PGC-1α‘s effects [81]. This synergy between Sirt3 and AMPK underscores their collective role in adjusting cellular metabolism to meet energy demands, especially during periods of caloric restriction and increased physical activity.

Moreover, Sirt3 demonstrates a protective role in the cellular response to stress. Mitochondria are often the first responders to cellular stress, and Sirt3’s involvement in regulating stress responses is paramount. By activating transcription factors such as forkhead box O3 (FOXO3a) and maintaining mitochondrial DNA integrity, Sirt3 enhances cellular resilience against metabolic and oxidative stress [82,83]. This stress resilience is crucial for cell survival and longevity, and it exemplifies the diverse regulatory mechanisms employed by Sirt3 to safeguard mitochondrial health. In terms of mitochondrial DNA maintenance, Sirt3 represents a preventative and reparative function. It assists in regulating enzymes key to mitochondrial DNA replication and repair [84]. For instance, the association between Sirt3 and the human 8-oxoguanine DNA glycosylase (OGG1) ensures the repair of oxidative DNA lesions and maintains mitochondrial genomic stability [85]. This ability to guard against mitochondrial DNA damage is particularly significant in mitigating the effects of aging and disease. In conclusion, Sirt3’s role in mitochondrial biogenesis is many-sided, involving the activation and regulation of critical pathways and transcription factors such as PGC-1α and AMPK, enhancing cellular stress responses, and maintaining mitochondrial DNA integrity. By modifying these processes, Sirt3 supports the formation of new, healthy mitochondria essential for cellular energy production and metabolic flexibility.

## 7. Future Research Directions

As discussed in the previous sections, SIRT3’s impact on the development and maturation of oocytes extends far beyond these early stages, impacting the broader function of oocytes post-fertilization and influencing subsequent embryonic development. Upon fertilization, the maturation-promoting functions of SIRT3 continue to yield benefits, which become evident in the subsequent stages of embryonic development. SIRT3 additionally influences maternal metabolism and gestational outcomes, with some evidence suggesting it might affect placental function and fetal health [86,87]. Though precise mechanistic links still remain elusive, the role of SIRT3 in these broader aspects of maternal-fetal medicine remains an intriguing area for further exploration. The clinical implications of SIRT3 modulation are compelling, particularly in enhancing oocyte quality and extending reproductive longevity. The ability to upregulate SIRT3 to maintain mitochondrial integrity and antioxidative defense mechanisms offers potential therapeutic interventions for tackling age-related declines in oocyte quality and mitigating the impacts of metabolic disorders like obesity and PCOS on fertility. Enhancing SIRT3 activity could open novel therapeutic strategies for improving oocyte resilience and performance and, by this means, address critical challenges in assisted reproductive technologies. Research has also shown that targeting SIRT3 could help in the development of fertility-preserving treatments, particularly for individuals undergoing gonadotoxic therapies such as chemotherapy [88]. The adaptive response of SIRT3 to gonadotoxic damage accentuates its potential role in preserving oocyte function and preventing premature ovarian failure. For example, interventions using melatonin and curcumin to modulate SIRT3 expression have demonstrated protective effects against oxidative damage, hinting at broader applications for these compounds in clinical settings [89]. Despite these promising insights, several critical gaps in understanding remain. One significant area of inquiry is the interaction between SIRT3 and other mitochondrial sirtuins, such as SIRT4 and SIRT5, particularly their antagonistic or cooperative signaling mechanisms. The complexity of the mitochondrial sirtuin network requires further investigation to untangle these interactions and understand their collective impact on oocyte health and mitochondrial function [90]. Additionally, the role of SIRT3 in responding to exogenous stressors such as cryopreservation and the handling of oocytes during in vitro fertilization (IVF) processes warrants further exploration. Understanding how these factors influence SIRT3 levels could lead to improved protocols that enhance oocyte viability and developmental potential post-thaw. Similarly, more research is needed to elucidate the epigenetic modifications that SIRT3 might induce or be affected by, particularly in the context of lifestyle factors like high-fat diets and their transgenerational impact on fertility [91,92]. The potential therapeutic applications of SIRT3 modulation are diverse, ranging from dietary interventions to pharmacological agents. Current strategies being investigated include the use of naturally occurring compounds such as curcumin, melatonin, and dihydromyricetin, alongside interventions like calorie restriction, exercise, and the administration of nicotinamide mononucleotide [93]. These approaches aim to harness SIRT3’s regulatory prowess to enhance oocyte quality and reproductive outcomes, especially in populations facing reproductive challenges due to age or metabolic stress.

As previously mentioned, granulosa and cumulus cells are crucial in the development and maturation of the oocyte, the female reproductive cell. These somatic cells surrounding the oocyte provide essential support and nourishment for the oocyte, enabling it to reach its full developmental potential, which is especially important during reproductive aging [94]. Since cumulus cells present a more glycolytic phenotype, they can provide metabolic substrates that mammalian oocytes can use to produce ATP via oxidative phosphorylation. Moreover, oogenesis requires the proper communication of oocytes and surrounding granulosa and cumulus cells. Differentiated cumulus cells are essential for oocyte nuclear and cytoplasmic maturation. They provide the eggs with the nutrients and regulatory signals necessary for their further development [95]. One key factor involved in the function of these aforementioned cells is the protein SIRT3. In the context of granulosa and cumulus cells, SIRT3 has been shown to impact the regulation of these cells’ function and survival. Previous research provided strong evidence that SIRT3 is expressed in granulosa and cumulus cells, and its activity is essential for maintaining the health and developmental capacity of the oocyte [96]. Specifically, SIRT3 has been shown to protect granulosa cells from oxidative stress-induced apoptosis, a common challenge faced by these cells during follicular development. The importance of SIRT3 in granulosa and cumulus cells is further highlighted by the fact that its dysregulation has been associated with various reproductive disorders, such as polycystic ovarian syndrome and age-related infertility [97,98]. Interestingly, recent research has explored the potential of using mitochondrial treatments, such as those derived from menstrual blood-derived stem cells, to improve ovarian function and oocyte quality. These studies suggest that restoring mitochondrial function, potentially through the modulation of SIRT3 activity, could be a promising avenue for enhancing the developmental capacity of in vitro-matured oocytes and addressing age-related decline in female fertility [99]. In a recent study, it has been determined that a lowered expression of the histone acetyltransferase 1 (HAT1), which is, among others, involved in the aging process of different organs, in granulosa cells is a potential reason that corresponds to the meiotic defects in oocytes of advanced age donors [100]. In summary, the exploration of SIRT3 in oocyte function beyond its development and maturation opens numerous avenues for clinical interventions to enhance female reproductive health and reduce the effects of aging on oocytes and surrounding cells. This chapter has synthesized existing findings and outlined significant future research directions, underscoring the critical role of SIRT3 in oocyte biology and its potential for therapeutic interventions to improve fertility and reproductive longevity. Comprehensive exploration and targeted research on SIRT3 will continue to illuminate its multifaceted contributions to reproductive success and inform the development of innovative treatments for infertility.

## 8. Conclusions

The study of SIRT3 has unveiled its remarkable significance in the realm of reproductive biology, particularly in the context of oocyte development, maturation, and function. Through a series of comprehensive analyses, this commentary has synthesized the existing knowledge on SIRT3, providing a cohesive understanding of its multifaceted roles and potential implications for fertility treatments. SIRT3’s influence on oocyte development is undeniable, comprising the modulation of mitochondrial function, metabolic processes, and oxidative stress responses. By enhancing the activity of key enzymes such as superoxide dismutase 2 (SOD2) through deacetylation, SIRT3 ensures the maintenance of a delicate redox balance, indispensable for the health and viability of oocytes (“Mitochondrial Sirtuins in Reproduction—Semantic Scholar”). Its dynamic expression, modulated by physiological and environmental factors, further underlines its importance in adapting to metabolic challenges like obesity and polycystic ovary syndrome (PCOS). Delving into oocyte maturation, SIRT3 has emerged as a critical player in each stage, from germinal vesicle breakdown to metaphase II arrest. Its regulatory roles extend to the SIRT3-GSK3β deacetylation pathway, which influences metabolic homeostasis, and the PGC-1α/SIRT3 axis, integral to mitochondrial biogenesis and antioxidative defense. Experimental evidence from Sirt3-null mice and various intervention studies underscores SIRT3’s indispensable role in safeguarding mitochondrial integrity against oxidative damage, thus promoting successful oocyte maturation and quality. The potential clinical implications of SIRT3 modulation are extensive. By targeting SIRT3 activity, it may be possible to enhance oocyte quality, extend reproductive longevity, and mitigate the detrimental effects of age and metabolic disorders on fertility. Interventions with compounds like melatonin, curcumin, and dihydromyricetin show promise in harnessing SIRT3’s regulatory capabilities for therapeutic benefits. Moreover, its relevance in counteracting gonadotoxic damage suggests its potential applications in fertility preservation for cancer survivors. However, despite this progress, several gaps remain in our understanding of SIRT3’s complete range of functions and interactions. The interplay between SIRT3 and other mitochondrial sirtuins, the impact of cryopreservation on SIRT3 levels, and the epigenetic modifications influenced by SIRT3 are areas ripe for further research. Addressing these gaps could unveil novel strategies to optimize oocyte development and fertility outcomes through targeted SIRT3 modulation. In conclusion, SIRT3 stands out as a central figure in ensuring oocyte health and reproductive success. Its comprehensive regulatory roles and adaptability to various physiological states highlight its potential as a therapeutic target. Continued research into SIRT3’s mechanisms and applications holds promise for advancing the field of reproductive medicine, ultimately contributing to improved fertility treatments and better clinical outcomes for individuals facing reproductive challenges.

## Data Availability

Not applicable.
